# Visual impairment and spectacle coverage rate in Baoshan district, China: population-based study

**DOI:** 10.1186/1471-2458-13-311

**Published:** 2013-04-08

**Authors:** Mengjun Zhu, Xiaowei Tong, Rong Zhao, Xiangui He, Huijuan Zhao, Meiling Liu, Jianfeng Zhu

**Affiliations:** 1 Shanghai Eye Disease Prevention and Treatment Center, No.380, Kangding Road, Jingan, Shanghai, 20040, China; 2 Center of Disease Control and Prevention of Baoshan District, No.158, Yueming Road, Baoshan, Shanghai, 201901, China; 3 Department of Epidemiology, College of Public Health, Fudan University, No.138, Yixueyuan Road, Xuhui, Shanghia, 200032, China

## Abstract

**Background:**

To investigate the prevalence and risk factors of visual impairment associated with refractive error and the unmet need for spectacles in a special suburban senior population in Baoshan District of Shanghai, one of several rural areas undergoing a transition from rural to urban area, where data of visual impairment are limited.

**Methods:**

The study was a population based survey of 4545 Chinese aged (age: >60 years or older ) at Baoshan, Shanghai, in 2009. One copy of questionnaire was completed for each subject. Examinations included a standardized refraction and measurement of presenting and best corrected visual acuity (BCVA) as well as tonometry, slit lamp biomicroscopy, and fundus photography.

**Results:**

The prevalence of mild (6/12 to 6/18), moderate (6/18 to 6/60) and severe visual impairment was 12.59%, 8.38% and 0.44%, respectively, and 5.26%, 3.06% and 0.09% with refractive correction. Visual impairment was associated with age, gender, education and career, but not insurance . The prevalence of correctable visual impairment was 5.81% (using 6/18 cutoff) and 13.18% (using 6/12 cutoff). Senior people and women were significantly at a higher risk of correctable visual impairment, while the well-educated on the contrary. The prevalence of undercorrected refractive error (improves by 2 or more lines with refraction) was 24.84%, and the proportion with undercorrected refractive error for mild, moderate , severe and no visual impairment was 61.54%, 67.98%, 60.00% and 14.10%, respectively. The spectacle coverage rate was 44.12%. Greater unmet need for spectacles was observed among elderly people, females, non-peasant, and subjects with less education and astigmatism only.

**Conclusions:**

High prevalence of visual impairment, visual impairment alleviated by refractive correction, and low spectacle coverage existed among the senior population in Baoshan District of Shanghai. Education for the public of the importance of regular examination and appropriate and accessible refraction service might be helpful to solve the problem.

## Background

Blindness and visual impairment represents a severe public health, social, and economic problem worldwide [1-4], while refractive error, a remediable cause of visual impairment [5], is one of the five priorities of the global initiative for elimination of avoidable blindness [6]. However, visual impairment caused by refractive error has received little attention. The reason probably is visual impairment usually is reported only after refractive correction according to the definition of WHO’s visual impairment categories,which refers to BCVA in better eye rather than presenting visual acuity [5].

Recently, several population-based studies in China [7-9] have examined associations between refractive error and visual impairment. All these studies emphasized that undercorrected refractive error was one of the most common causes of visual impairment. The Tehran Eye Study [10] showed that the visual acuity of 26.7% subjects was improved of at least 1 line, and 5.9% even of ≥ 4 lines via proper correction. Furthermore, both individual and social costs of visual impairment due to refractive error are higher than that of cataract due to the early onset age of the former [11,12].

As a result of China’s rapid economic growth, many rural areas in the country are experiencing progressive urbanization. Although acknowledge of the prevalence and causes of visual impairment at a regional level is essential for developing local policies for optimal utilization of medical resource to eliminate avoidable visual impairment [13], data of such characteristic regions are limited. Therefore, we investigated the prevalence and risk factors of visual impairment as well as unmet needs for spectacles and associated factors among senior population aged 60 or above in Baoshan District, which is one of several rural areas of Shanghai and a typical representative of such regions. Selecting elderly people as study population was mainly because Shanghai has reached an aging society [14], and also was motivated by several considerations. Firstly, the prevalence of visual impairment is relatively high in elder population [9]. Secondly, most of people ≥ 60y in Shanghai have retired and their enumeration can be easily obtained from official resident register compared with young-migrating people.

## Methods

### Material and methods

The BaoShan Eye Study was a population-based, cross-sectional survey of eye diseases among 4545 Chinese aged ≥60 years in Dachang County of Baoshan District, which is one of 18 districts of Shanghai, China. The population of Baoshan District was 864, 346 by December 31, 2009, of which Dachang country accounted for 15.33% (132, 479) [15]. Compared to other districts in Shanghai, Baoshan District has a low-to-middle level medical services which parallels to the income of its residents. With the process of urbanization, the proportion of elderly population and level of education in Baoshan District slightly increased compared to those of 2000 [16]. The current investigation followed the tenets of the Declaration of Helsinki and was approved by Ethical Committee of Shanghai Eye Disease Prevention and Treatment Center. All participants signed written informed consent before participating in the study. Subjects aged ≥60 years were selected using a randomized, clustered sampling process. All subjects were derived from Dachang County of Baoshan District.

### Sampling procedure and participants

A random cluster sampling technique was used. Based on previous studies, the prevalence of visual impairment was anticipated to be 4.00% and the allowable error bound to be 20% with 95% confidence levels [9,17,18]. An 85% response rate and a design effect of 1.5 were used to calculate the requisite sample size of 4068 (according to the formula N = Z^2^ (p) (1-p)/B^2^ (p = 0.0265, B = 0.0265 × 0.25, Z = 1.96). According to 2009 census data, Dachang county of Baoshan District had a population of 132, 479 [19], among which 22.0% were 60 years old or above [20]. Vacant households, residents who died before contact and inpatient residents were excluded; thus 108, 469 subjects actually participated in the study. We defined a population of approximately 1000 individuals as the basic sample unit (BSU) and a total of 108 BSU were included. Each standard BSU has 220 subjects with an age of >60 years old. Based on the calculated minimal sampling number of 4068, the theoretical sampling unit was 19. Our preliminary studies showed that the population with an age of >60 years old accounted for less than 22%, a total of 30 BSN were actually selected from the investigating area (total 10 neighborhoods) in the sampling frame to ensure the study size. All BSU was numbered and randomly selected from the investigating area. A total of 5199 individuals were eligible. Door-to-door visits were conducted by trained enumerators at all households within the sample frame. The whole procedure and purpose of this study were explained and finally 4545 (87.42%) subjects participated in our study.

### Enumeration and examination procedures

Local hospitals were chosen as examination sites. Trained eye health care workers filled out a detailed questionnaire investigating on socioeconomic background, education level, occupation, insurance, living conditions, smoking and alcohol consumption, intake of medication, psychiatric status, wearing and availability of glasses, family history of eye diseases, and the presence of any eye abnormalities with details. Standardized ophthalmic examinations beginning with visual acuity (VA) test were performed by ophthalmologists, optometrists and technicians. Presenting VA (with spectacles if worn), and VA after refractive correction were measured using logMAR chart with tumbling E at a distance of 4 m. Autorefraction (Topcon KR-8900, Japan) was performed for all subjects independent of the VA. Subjective refraction exam was performed only for those with VA of worse than 16/20. VA was recorded as the smallest line recognized with one or no error. If the top line cannot be recognized at 4 m, the participant was moved to 3, 2, or 1 m, consecutively. If the participant cannot recognize the top line at 1 meter yet, VA was assessed by counting fingers, hand movements, and perception of light. For physically disabled people who could not come to examination sites, ocular examinations (using portable equipment) in house were provided. As autorefraction cannot be performed in house, retinoscopy and subjective refraction were used to obtain their BCVA. Since the study was to understand the causes for visual impairment and the corresponding eye disease screening, we did not investigate the near vision and its correction of the subjects. In addition, slit-lamp biomicroscopy exams were performed by ophthalmologists, and the abnormalities in anterior segment were noted if any. Intraocular pressure was measured via a non-contact tonometer (Canon TX-F, Japan). If IOP was higher than 21 mmHg, the measurement was repeated. Digital monoscopic photographs of the optic disc and macula were obtained (Canon CR6-45 nm, Japan). All fieldwork was conducted from October 2009 to December 2009.

### Definitions

World Health Organization categories of vision loss were used to define blindness and severe visual impairment [21]. For mild and moderate visual impairment, a similar definition was used which has been published in previous studies [13,22-24].

Blindness: A presenting VA (with glasses for distance if normally worn or unaided if glasses for distance not worn) of <3/60 in the better eye.

Severe Visual Impairment (SVI): A presenting VA of <6/60 to 3/60 in the better eye.

Moderate Visual Impairment (Mod VI): A presenting VA of <6/18 to 6/60 in the better eye.

Mild Visual Impairment (Mild VI): A presenting VA of <6/12 to 6/18 in the better eye.

Normal (N): A presenting VA of ≥ 6/12 in the better eye.

Correctable visual impairment was defined as presenting VA (naked eye if without spectacles and with distance eyeglasses if worn) in the better eye of 6/18 that improved to no impairment (≥ 6/18) after refractive correction. The cutoff of 6/12 was regarded as another standard of the correctable visual impairment for more convenient comparison [8].

The less stringent Victoria Visual Impairment Project (VVIP) defined undercorrected refractive error as individuals with presenting VA worse than 20/20 minus 2 letters which improved by five letters or more (1 line or more of visual acuity) in the better eye after refraction [25]. Considering the need of comparison with other study [26], we used the definition of undercorrected refractive error as an improvement of two or more lines in visual acuity in the better eye after refraction of the same eye.

We used the definitions presented by Bourne and colleagues to determine the spectacle coverage [10]. “Met need” for spectacles was defined as the number of subjects who wore spectacles and had visual acuity worse than 20/40 in the better eye without correction, but achieved 20/40 or better with their present spectacles. The methodology used in our survey tested VA with the subjects habitual distance correction. A bespectacled subject was not retested without wearing spectacles at the time of presentation. Therefore, for the purposes of this survey, it was assumed that spectacle wearers would have acuity worse than 6/12 in the better eye without spectacles. “Unmet need” was defined as the number of subjects who had a visual acuity worse than 20/40 in the better eye without correction and could achieve 20/40 or better in the better eye with correction, but did not wear spectacles or did not achieve such correction with their present spectacles. With these definitions, spectacle coverage was calculated as: spectacle coverage(%)= Met need *100%/(Met need + Unmet need).

The refractive data were converted to spherical equivalent (SE) for our definitions of myopia, hyperopia. Myopia was defined as an SE of less than −0.5 D. For further analysis, myopia was classified in three groups: mild (−0.5 to −3.0D), moderate (>−3.0 to −6.0D), and severe (>−6.0D). Hyperopia was defined as an SE of >0.5D, and also classified into three groups: mild (+0.5 to +2.0D), moderate (>2.0 to +4.0D) and severe (>+4.0D). Simple astigmatism was defined as cylinder of greater than 0.5 D and spherical between −0.50D and +0.50D. Since the classification is only used in calculation of spectacle coverage, we included the refractive results of the better eyes.

### Statistical methods

All data were analyzed by commercially available statistical software (SPSS for Windows version 16.0), 95% Confidence intervals (CIs) and *P*-values (significant at the *P*<0.05 level) were calculated for the estimation of prevalence. Odds ratios (OR; presented with 95% confidence intervals) were used in univariate analysis of visual impairment, correctable visual impairment and unmet need with key variables, such as age, gender, education, occupation and insurance.

Multiple logistic regression analysis was used to fit the best model for independent variables (all the variables analyzed in univariate analysis were included in multivariate models) in order to determine the predictive factors for visual impairment, correctable visual impairment and unmet need, respectively. Population prevalence rates of visual impairment and correctable visual impairment were also calculated by direct age standardization to the 2000 population census of Shanghai [27].

## Results

In this study, 641 of the 5199 subjects aged≥60 years refused to participate in the examination and 13 were excluded of the remaining due to mental retardation, deaf, and mental disorders. Thus a total of 4545 were examined with the response rate 87.42% (95% CI, 86.52%–88.32%). Among 4545 subjects, 57.96% (2635/4545) were female. The mean age of all examined subjects was 68.40±8.29 years old (SD). The age of males (68.78±8.02) was significantly higher that of females (68.12±8.48; t=2.704, *P*=0.007). Among the 654 non-participants, elderly (72.00±8.22 years vs.68.40±8.29 years, t=10.400, *P*<0.001) and male(χ^2^=29.12, *P*<0.001)were less likely to participate.

Among the 4545 subjects with presenting VA, the overall prevalence of mild, moderate and severe visual impairment was 12.59% (95% CI, 11.62%–13.55%), 8.38% (95% CI, 7.58%–9.19%) and 0.44% (95% CI, 0.25%–0.63%), respectively. The occurrence of subjects presenting blindness was 0.86% (95% CI, 0.59%–1.13%). With best-corrected VA, the prevalence of mild, moderate and severe visual impairment was 5.26% (95% CI, 4.61%–5.91%), 3.06% (95% CI, 2.56%–3.56%) and 0.09% (95% CI, 0.00%–0.17%), respectively. Blindness with best correction was 0.66% (95% CI, 0.42%–0.90%). After refractive correction, the prevalence of mild, moderate and severe visual impairment was significantly decreased(mild:χ^2^=150.13, *P*<0.001, moderate:χ^2^=119.46, *P*<0.001, severe:χ^2^=10.69, *P*=0.001), while the prevalence of blindness was not significantly changed (χ^2^=1.82, P=0.277). For the female subjects presenting VA, the prevalence of mild, moderate and severe visual impairment was 14.69%, 9.53% and 0.61%, respectively. For the male subjects presenting VA, the prevalence of mild, moderate and severe visual impairment was 9.69%, 6.81% and 0.21%, respectively. The prevalence of mild visual impairment in females was significantly higher than that in males (*P*<0.001). In other groups of visual impairment, the difference caused by gender was not significant (*P*>0.05). Similar results were obtained in the subjects with best-corrected VA (Table 1).

**Table 1:** Prevalence rates of blindness, SVI, Mod VI and Mild VI by age, sex, insurance, occupation and education*

**Characteristic**		**Presenting visual acuity**				**Best-corrected visual acuity**			
	**Total n**	**Blind n(%;95%CI)**	**SVI n(%;95%CI)**	**Mod VI n(%;95%CI)**	**Mild VI n(%;95%CI)**	**Blind n(%;95%CI)**	**SVI n(%;95%CI)**	**Mod VI n(%;95%CI)**	**Mild VI n(%;95%CI)**
**Age,y**									
60-69	2907	14(0.48;0.23-0.73)	7(0.24;0.06-0.42)	152(5.22;4.42-6.04)	211(7.26;6.32-8.20)	10(0.34;0.13-0.56)	1(0.03;0–0.10)	37(1.27;0.87-1.68)	61(2.10;1.58-2.62)
70-79	1103	7(0.63;0.17-1.10)	6(0.54;0.11-0.98)	112(10.15;8.37-11.94)	201(18.22;15.94-20.50)	6(0.54;0.11-0.98)	2(0.18;0–0.43)	35(3.17;2.14-4.21)	87(7.89;6.30-9.48)
80~	535	18(3.36;1.84-4.89)	7(1.31;0.35-2.27)	117(21.87;18.37-25.37)	160(29.91;26.03-33.79)	14(2.62;1.26-3.97)	1(0.19;0–0.55)	67(12.52;9.72-15.33)	91(17.01;13.83-20.19)
total	4545	39(0.86;0.59-1.13)	20(0.44;0.25-0.63)	381(8.38;7.58-9.19)	572(12.59;11.62-13.55)	30(0.66;0.42-0.90)	4(0.09;0.00-0.17)	139(3.06;2.56-3.56)	239(5.26;4.61-5.91)
Age adjusted**†**		0.89(0.62-1.16)	0.48(0.28-0.68)	9.05(8.22-9.89)	14.02(13.01-15.03)	0.69(0.45-0.93)	0.11(0.01-0.20)	3.34(2.81-3.86)	6.03(5.34-6.72)
**Gender**									
Male	1910	17(0.89;0.47-1.31)	4(0.21;0-0.41)	130(6.81;5.68-7.94)	185(9.69;8.36-11.01)	13(0.68;0.31-1.05)	1(0.05;0–0.15)	47(2.46;1.77-3.16)	68(3.56;2.73-4.39)
Female	2635	22(0.83;0.49-1.18)	16(0.61;0.31-0.90)	251(9.53;8.40-10.65)	387(14.69;11.34-16.04)	17(0.65;0.34-0.95)	3(0.11;0–0.24)	92(3.49;2.79-4.19)	171(6.49;5.55-7.43)
**Type of insurance**									
No insurance	20	0(0;0-17.00)	0(0;0-17.00)	2(10.00;1.00-32.00)	2(10.00;1.00-32.00)	0(0;0–17.00)	0(0;0–17.00)	0(0;0–17.00)	1(5.00;0–24.00)
Urban insurance	2271	19(0.84;0.46-1.21)	11(0.48;0.20-0.77)	208(9.16;7.97-10.35)	284(12.51;11.15-13.87)	17(0.75;0.39-1.10)	1(0.04;0–0.13)	70(3.08;2.37-3.79)	127(5.59;4.65-6.54)
Others	2254	20(0.89;0.50-1.27)	9(0.40;0.14-0.66)	171(7.59;6.49-8.68)	286(12.69;11.31-14.06)	13(058;0.26-0.89)	3(0.13;0–0.28)	69(3.06;2.35-3.77)	111(4.92;4.03-5.82)
**Occupation**									
Peasant	1232	14(1.14;0.54-1.73)	4(0.32;0.01-0.64)	85(6.90;5.48-8.314)	156(12.66;10.81-14.52)	10(0.81;0.31-1.31)	1(0.08;0–0.24)	31(2.52;1.64-3.39)	61(4.95;3.74-6.16)
Non-peasant	3313	25(0.75;0.46-1.05)	16(0.48;0.25-0.72)	296(8.93;7.96-9.91)	416(12.56;11.43-13.68)	20(0.60;0.34-0.87)	3(0.09;0–0.19)	108(3.26;2.66-3.86)	178(5.37;4.60-6.14)
**Level of Education**									
Illiteracy	684	14(2.05;0.99-3.11)	4(0.58;0.01-1.16)	100(14.62;11.97-17.27)	149(21.78;18.69-24.88)	10(1.46;0.56-2.36)	1(0.15;0–0.43)	50(7.31;5.36-9.26)	78(11.40;9.02-13.79)
Primary school	2319	14(0.60;0.29-0.92)	14(0.60;0.29-0.92)	186(8.02;6.92-9.13)	280(12.07;10.75-13.40)	12(0.52;0.23-0.81)	3(0.13;0–0.28)	62(2.67;2.02-3.33)	113(4.87;4.00-5.75)
Secondary school or higher	1542	11(0.71;0.29-1.13)	2(0.13;0-0.31)	95(6.16;4.96-7.36)	143(9.27;7.83-10.72)	8(0.52;0.16-0.88)	0(0;0–17.00)	27(1.75;1.10-2.41)	48(3.11;2.25-3.98)

*Data are given as numbers of persons (prevalence percentage; 95% confidence interval).

**†**Age adjusted to the 2000Shanghai census population. Data are given as prevalence percentage (95% confidence interval).

According to presenting visual acuity, 973 (21.41%) of the 4545 subjects had visual impairment for a 6/12 cutoff. After refractive correction, only 374 subjects (8.23%) still had visual impairment. For a 6/18 cutoff, these values were 401 and 137, respectively. The prevalence of correctable visual impairment was 13.18% (95% CI, 12.20%–14.16%) for a 6/12 cutoff and 5.81% (95% CI, 5.13%–6.49%) for a 6/18 cutoff. Of the 599 subjects with correctable visual impairment for a 6/12 cutoff, only 98 (16.36%) wore distance spectacles, while the proportion was 18.94% (50/264) for a 6/18 cutoff (Table 2). The prevalence of visual impairment and correctable visual impairment for a 6/18 cutoff was higher in the subjects with older age and less education (*P*<0.01). The prevalence of visual impairment and correctable visual impairment were significantly higher in females than in males (*P*<0.01).

**Table 2:** Prevalence rates of VI, correctable VI and noncorrectable VI for two criteria by age, sex, insurance, occupation and education*

**Characteristic**	**Presenting visual acuity for a 6/18 cutoff**			**Presenting visual acuity for a 6/12 cutoff**		
	**VI n(%;95%CI)**	**Correctable VI n(%;95%CI)**	**Noncorrectable VI n(%;95%CI)**	**VI n(%;95%CI)**	**Correctable VI n(%;95%CI)**	**Noncorrectable VI n(%;95%CI)**
**Age,y**						
60-69	159(5.47;4.64-6.30)	124(4.27;3.53-5.00)	35(1.20;0.81-1.60)	370(12.73;11.52-13.94)	274(9.43;8.36-10.49)	96(3.30;2.65-3.95)
70-79	118(10.70;8.87-12.52)	82(7.43;5.89-8.98)	36(3.26;2.22-4.31)	319(28.92;26.25-31.60)	196(17.77;15.51-20.03)	123(11.15;9.29-13.01)
80~	124(23.18;19.60-26.75)	58(10.84;8.21-13.48)	66(12.34;9.55-15.12)	284(53.08;48.86-57.31)	129(24.11;20.49-27.74)	155(28.97;25.13-32.82)
total	401(8.82;8.00-9.65)	264(5.81;5.13-6.49)	137(3.01;2.52-3.51)	973(21.41;20.22-22.60)	599(13.18;12.20-14.16)	374(8.23;7.43-9.03)
Age adjusted**†**	9.53(8.18-10.39)	6.22(5.52-6.93)	3.31(2.79-3.83)	23.55(22.32-24.79)	14.26(13.25-15.28)	9.30(8.45-10.14)
**Gender**						
Male	134(7.02;5.87-8.16)	89(4.66;3.71-5.60)	45(2.36;1.68-3.04)	319(16.70;15.03-18.37)	207(10.84;9.44-12.23)	112(5.86;4.81-6.92)
Female	267(10.13;8.98-11.29)	175(6.64;5.69-7.59)	92(3.49;2.79-4.19)	654(24.82;23.17-26.50)	392(14.88;13.52-16.24)	262(9.94;8.80-11.09)
**Type of insurance**						
No insurance	2(10.00;1.00-32.00)	2(10.00;1.00-32.00)	0(0;0–17.00)	4(20.00;6.00-44.00)	3(15.00;3.00-38.00)	1(5.00;0–25.00)
Urban insurance	219(9.64;8.43-10.86)	149(6.56;5.54-7.58)	70(3.08;2.37-3.79)	503(22.15;20.44-23.86)	306(13.47;12.07-14.88)	197(8.67;7.52-9.83)
Others	180(7.99;6.87-9.10)	113(5.01;4.11-5.91)	67(2.97;2.27-3.67)	466(20.67;19.00-22.35)	290(12.87;11.48-14.25)	176(7.81;6.70-8.92)
**Occupation**						
Peasant	89(7.22;5.78-8.67)	59(4.79;3.60-5.98)	30(2.44;1.57-3.30)	245(19.89;17.66-22.12)	156(12.66;10.81-14.52)	89(7.22;5.78-8.67)
Non-peasant	312(9.42;8.42-10.41)	205(6.19;5.37-7.01)	107(3.23;2.63-3.83)	728(21.97;20.56-23.38)	443(13.37;12.21-14.53)	285(8.60;7.65-9.56)
**Level of Education**						
Illiteracy	104(15.20;12.51-17.90)	55(8.04;6.00-10.08)	49(7.16;5.23-9.10)	253(36.99;33.37-40.61)	128(18.71;15.79-21.64)	125(18.27;15.38-21.17)
Primary school	200(8.62;7.48-9.77)	137(5.91;4.95-6.87)	63(2.72;2.06-3.38)	480(20.70;19.05-22.35)	304(13.11;11.74-14.48)	176(7.59;6.51-8.67)
Secondary school or higher	97(6.29;5.08-7.50)	72(4.67;3.62-5.72)	25(1.62;0.99-2.25)	240(15.56;13.75-17.37)	167(10.83;9.28-12.38)	73(4.73;3.67-5.79)

*Data are given as numbers of persons (prevalence percentage; 95% confidence interval).

**†**Age adjusted to the 2000 Shanghai census population. Data are given as prevalence percentage (95% confidence interval).

The subjects with older age were significantly prone to have visual impairment (≥80 years; OR, 8.31; 95% CI, 6.77–10.20 versus 60–69 years, 70–79 years; OR, 2.80; 95% CI, 2.36–3.32 versus 60–69 years). The lowest prevalence of visual impairment for a 6/12 cutoff was observed in the subjects from 60 to 69 years old (12.73%), while the highest prevalence was observed in the subjects more than 80 years old(53.08%). Females had a significant higher risk of visual impairment (OR 1.65, 95%CI 1.42-1.91) than males. A higher level of education was a protective factor for visual impairment (Junior high school or higher; OR, 0.31; 95% CI, 0.25–0.38 versus No education, Primary school; OR, 0.43; 95% CI, 0.36–0.52 versus No education). Career and the types of insurance were not significantly associated with the risk of visual impairment. In last multivariate logistic modeling, all variables, except insurance, were significantly associated with visual impairment (Table 3).

**Table 3:** Univariate and multivariate analysis of visual impairment and correctable visual impairment for a 6/12 cutoff

**Characteristic**	**No VI**	**VI**			**Correctable VI**		
	**n**	**n**	**Univariate odds ratio (95% CI)**	**Multivariate odds ratio (95% CI)**	**n**	**Univariate odds ratio (95% CI)**	**Multivariate odds ratio (95% CI)**
**Age,y**							
60-69	2523	370	1.00(reference)	1.00(reference)	274	1.00(reference)	1.00(reference)
70-79	777	319	2.80***(2.36-3.32)	2.62***(2.19-3.13)	196	2.32***(1.90-2.84)	2.24***(1.82-2.76)
80~	233	284	8.31***(6.77-10.20)	7.48***(6.00-9.33)	129	5.10***(3.98-6.54)	4.80***(3.69-6.26)
**Gender**							
Male	1574	319	1.00(reference)	1.00(reference)	207	1.00(reference)	1.00(reference)
Female	1959	654	1.65***(1.42-1.91)	1.67***(1.42-1.97)	392	1.52***(1.27-1.82)	1.56***(1.29-1.88)
**Type of insurance**							
No insurance	16	4	1.00(reference)	1.00(reference)	3	1.00(reference)	1.00(reference)
Urban insurance	1749	503	1.15(0.38-3.46)	1.24(0.38-4.06)	306	0.93(0.27-3.22)	1.02(0.28-3.72)
Others	1768	466	1.05(0.35-3.17)	1.02(0.31-3.34)	290	0.88(0.25-3.02)	0.88(0.24-3.21)
**Occupation**							
Peasant	973	245	0.89(0.75-1.04)	0.73***(0.60-0.89)	156	0.93(0.76-1.13)	0.80(0.64-1.01)
Non-peasant	2560	728	1.00(reference)	1.00(reference)	443	1.00(reference)	1.00(reference)
**Level of education**							
Illiteracy	417	253	1.00(reference)	1.00(reference)	128	1.00(reference)	1.00(reference)
Primary school	1825	480	0.43***(0.36-0.52)	0.74**(0.60-0.93)	304	0.54***(0.43-0.69)	0.81(0.62-1.06)
Secondary school or higher	1291	240	0.31***(0.25-0.38)	0.55***(0.42-0.71)	167	0.42***(0.33-0.54)	0.65**(0.48-0.89)

CI=confidence interval.

* Significant at P <0.05.

** Significant at P <0.01.

*** Significant at P <0.001.

Table 3 showed the association between correctable visual impairment and various independent variables. Old age (≥80 years; OR, 5.10; 95% CI, 3.98–6.54 versus 60–69 years, 70–79 years; OR, 2.32; 95% CI, 1.90–2.84 versus 60–69 years) and female gender (OR, 1.52; 95% CI, 1.27 –1.82) were significantly associated with correctable visual impairment for a 6/12 cutoff . On the other hand, a higher level of education (Junior high school or higher; OR, 0.42; 95% CI, 0.33–0.54 versus No education, Primary school; OR, 0.54; 95% CI, 0.43–0.69 versus No education) was a protective factor for correctable visual impairment.

In the final multiple logistic regression analysis controlling for all covariates, older age (≥80 years; OR, 4.80; 95% CI, 3.69–6.26 versus 60–69 years, 70-79 years; OR,2.24; 95% CI, 1.82–2.76 versus 60–69 years) and female gender (OR 1.56, 95%CI 1.29-1.88) were significantly associated with correctable visual impairment. A higher level of education, e.g., junior high school or higher, (OR, 0.65; 95% CI, 0.48–0.89) was a protective factor for correctable visual impairment. Career and the types of insurance showed no significant association with correctable visual impairment.

Table 4 showed the improvement in the participants’ vision by correcting their undercorrected refractive errors. Utilization of appropriate spectacles improved the visual acuity by at least one line in 45.43% (95%CI, 43.99% to 46.88%) of the studied population and as high as four lines or more in 5.79% (95%CI, 5.11% to 6.47%). These figures were more pronounced among participants with visual impairment, especially in severe/moderate visual impairment.

**Table 4:** Visual improvement after correcting refractive errors

		**Correctable**		**Uncorrectable**		**Total**	
	**Gained lines**	**n**	**%(95%CI)**	**n**	**%(95%CI)**	**n**	**%(95%CI)**
**SVI**		3		17		20	
	**0line**	0	0(0–71.00)	3	17.65(4.00-43.00)	3	15.00(3.00-38.00)
	**≥1 line**	3	100(29.00-100.00)	14	82.35(57.00-96.00)	17	85.00(62.00-97.00)
	**≥2 lines**	3	100(29.00-100.00	9	52.94(28.00-77.00)	12	60.00(36.00-81.00)
	**≥3 lines**	3	100(29.00-100.00)	8	47.06(23.00-72.00)	11	55.00(31.00-77.00)
	**≥4 lines**	3	100(29.00-100.00)	6	35.29(14.00-62.00)	9	45.00(23.00-69.00)
**Mod VI**		156		225		381	
	**0line**	0	0	82	36.44(30.16-42.73)	82	21.52(17.40-25.65)
	**≥1 line**	156	100(100.00-100.00)	143	63.56(57.27-69.84)	299	78.48(74.35-82.60)
	**≥2 lines**	156	100(100.00-100.00)	103	45.78(39.27-52.29)	259	67.98(63.29-72.66)
	**≥3 lines**	156	100(100.00-100.00)	71	31.56(25.48-37.63)	227	59.58(54.65-64.51)
	**≥4 lines**	136	87.18(81.93-92.43)	41	18.22(13.18-23.27)	177	46.46(41.44-51.46)
**Mild VI**		440		132		572	
	**0line**	0	0	88	66.67(58.62-74.71)	88	15.38(12.43-18.34)
	**≥1 line**	440	100(100.00-100.00)	44	33.33(25.29-41.38)	484	84.62(81.66-87.57)
	**≥2 lines**	352	80.00(76.26-83.74)	0	0	352	61.54(57.55-65.53)
	**≥3 lines**	193	43.86(39.23-48.50)	0	0	193	33.74(29.87-37.62)
	**≥4 lines**	73	16.59(13.11-20.07)	0	0	73	12.76(10.03-15.50)
**No VI**						3533	
	**0line**					2276	64.42(62.84-66.00)
	**≥1 line**					1257	35.58(34.00-37.16)
	**≥2 lines**					498	14.10(12.95-15.24)
	**≥3 lines**					92	2.60(2.08-3.13)
	**≥4 lines**					0	0
**Total**						4545	
	**0line**					2480	54.57(53.12-56.01)
	**≥1 line**					2065	45.43(43.99-46.88)
	**≥2 lines**					1129	24.84(23.58-26.01)
	**≥3 lines**					529	11.64(10.71-12.57)
	**≥4 lines**					263	5.79(5.11-6.47)

Among 572 participants (12.59%) who had mild visual impairment according to the aforementioned definition with a presenting visual acuity worse than 20/40 in the better eye, only 88 subjects (15.38%) had no improvement after correction, while 12.76% (95% CI, 10.03% to 15.50%) could experience a four line improvement by wearing proper spectacles. In addition, 46.46% of Mod VI (95% CI, 41.44% to 51.56%) and 45.00% of SVI (95% CI, 23.00% to 69.00%) obtained an improvement of at least four lines with accurately prescribed spectacles. Although correctable visual impairment gained more lines of improvement than uncorrectable visual impairment, 35.29% of uncorrectable SVI and 18.22% of uncorrectable Mod VI could gain four lines of improvement after refractive correction.

Of the 4545 participants, 1129 participants gained an improvement of two or more lines in visual acuity. Thus, the overall prevalence of undercorrected refractive error was 24.84% (95% CI, 23.58% to26.01%). If stratified by visual impairment (VI) categories, the prevalence of undercorrected refractive error for mild VI, moderate VI, severe VI and no VI were 61.54%, 67.98%, 60.00% and 14.10%, respectively (Table 4).

A total of 473 subjects accounting for 10.41% (95% CI, 9.52%–11.29%) were corrected for refractive error with spectacles (met need), while 599 subjects accounting for 13.18% (95% CI, 12.20%–14.16%) had unmet need. Among the unmet need, 501 subjects never wore spectacles or gave up wearing spectacles and the remaining 98 subjects wore an inappropriate spectacle. Thus spectacle coverage was 44.12%. Spectacle coverage declined with age (χ^2^=114.17, *P*<0.001), which was 56.99% in the youngest age group (60–69 years) and 15.69% in the oldest age group (≥80 years). In the contrary, the spectacle coverage was increased with education (χ^2^=133.18, *P*<0.001), which was 15.79% in the no-education group and 63.22% in the junior/high school education or higher group. Spectacle coverage was significantly lower in females (36.47%) than in males (54.51%) (χ^2^=34.56, *P*<0.001). In peasant and the subjects with insurance, the spectacle coverage was also significantly higher (χ^2^=62.74, P<0.001; χ^2^=11.51, *P*<0.01, respectively). The spectacle coverage in our study was significantly different in the subjects with different refractive errors (χ^2^=34.49, *P*<0.001). For example, the highest coverage was observed in myopia and the lowest coverage was in astigmatism. In myopia, spectacle coverage among moderate/high myopia was 64.62% and 53.44%, which was significantly higher than 33.96% in mild myopia (Moderate myopia vs. Mild myopia, χ^2^=44.39, *P*<0.001; High myopia vs. Mild myopia, χ^2^=13.81, P<0.001, respectively). In hyperopia, only mild hyperopia showed significant higher spectacle coverage than moderate hyperopia (48.66% in mild hyperopia vs.33.33% in moderate hyperopia, χ^2^=7.60, *P* = 0.006). No significant difference was observed between the coverage of the two remaining degree of hyperopia (*P*≥0.05) (Table 5).

**Table 5:** Spectacle coverage rate by sociodemographic variables as well as univariate and multivariate model of unmet need

**Characteristic**		**Total n**	**Met need (n)**	**Unmet need (n)**	**Spectacle coverage (%)**	**Univariate odds ratio (95% CI) of unmet need**	**Multivariate odds ratio (95% CI) of unmet need**
**Age,y**							
60-69		637	363	274	56.99	1.00(reference)	1.00(reference)
70-79		282	86	196	30.50	3.02^***^(2.24-4.07)	2.70^***^(1.91-3.82)
80~		153	24	129	15.69	7.12^***^(4.48-11.32)	5.82^***^(3.49-9.70)
Total		1072	473	599	44.12		
**Gender**							
Male		455	248	207	54.51	1.00(reference)	1.00(reference)
Female		617	225	392	36.47	2.09^***^(1.63-2.67)	2.04^***^(1.54-2.70)
**Occupation**							
Peasant		881	438	443	49.72	1.00(reference)	1.00(reference)
Non-peasant		191	35	156	18.32	4.41^***^(2.99-6.51)	2.82^***^(1.72-4.61)
**Type of insurance**							
No insurance		58	14	44	24.14	1.00(reference)	1.00(reference)
Urban insurance		541	235	306	43.44	0.41^**^(0.22-0.77)	1.67(0.79-3.56)
Others		473	224	249	47.36	0.35^***^(0.19-0.66)	1.29(0.61-2.73)
**Level of education**							
Illiteracy		152	24	128	15.79	1.00(reference)	1.00(reference)
Primary school		466	162	304	34.76	0.35^***^(0.22-0.57)	0.96(0.55-1.68)
Secondary school or higher		454	287	167	63.22	0.11^***^(0.07-0.18)	0.38^***^(0.21-0.67)
**Refractive error**							
**Myopia**		608	297	311	48.85	0.20^***^(0.12-0.36)	0.44^*^(0.23-0.83)
	Mild Myopia	265	90	175	33.96		
	Moderate Myopia	212	137	75	64.62		
	High Myopia	131	70	61	53.44		
**Hyperopia**		372	161	211	43.28	0.26^***^(0.14-0.46)	0.30^***^(0.16-0.57)
	Mild Hyperopia	224	109	115	48.66		
	Moderate Hyperopia	123	41	82	33.33		
	High Hyperopia	25	11	14	44.00		
**Astigmatism**		92	15	77	16.30	1.00(reference)	1.00(reference)
	Mild Astigmatism	27	4	23	14.81		
	High Astigmatism	65	11	54	16.92		

CI=confidence interval.

* Significant at P <0.05.

** Significant at P <0.01.

*** Significant at P <0.001.

Table 5 showed the association between unmet need and the various independent variables, e.g., age, gender, career, insurance, education and type of refractive error. Univariate analyses showed that all these variables were significantly associated with the participant′s unmet need. The adjusted effect of these variables on the unmet need was assessed by multivariate logistic regression analysis. In the final model, insurance showed no significant association with unmet need, while age, career, education (except for primary school), and type of refractive error had significant correlation with unmet need. Among these variables, higher educational level, myopia and hyperopia were protective factors for unmet need, while others were risk factors.

## Discussion

People with visual impairment are more likely to report difficulties associated with their daily activities [28,29]. For example, visual impairment, especially severe visual impairment, has been shown to be strongly associated with an increased risk for falling [30,31]. Refractive error is a significant cause of visual impairment. As a survey of visual impairment in elderly population, our study differed from previous surveys in China. Firstly, we included mild visual impairment as a category separate from normal vision. Secondly, the characteristic population is in the place undergoing urbanization. In our study, the prevalence of mild visual impairment was 12.95% with presenting vision and 5.26% after correction. The tendency was similar for moderate/severe visual impairment. If refractive correction had been available, the prevalence of moderate/severe visual impairment was reduced from 8.82% to 3.15%. According to one previous study which was conducted in 9 provinces of China, the prevalence of mild visual impairment ranged from 10.8% to 27.4% for the subjects with presenting vision and from 4.06% to 24.1% for the individuals with best correction [9]. At the same time, the prevalence of correctable visual impairment was 9.43% in our study, which was similar to 9.55% reported in Taiwan [8]. The fact that only 16.36% (98/599) of the correctable visual impairment for a 6/12 cutoff, and 18.94% (50/264) for a 6/18 cutoff had spectacles for distance correction indicated that refractive correction is still significantly underutilized, although a large number of visual impairment caused by refractive errors can easily be corrected by wearing the appropriate spectacles.

Our study indicated that 24.84% of residents in Baoshan district of Shanghai aged 60 years old and above had an undercorrected refractive error. The occurrence of undercorrected refractive error in this study was higher than previous studies, e.g., 10.2% in the Blue Mountains study [32], 15.1% in the Los Angeles Latino Eye Study [33], 17.3% in the Tanjong Pager Survey [26] and 20.4% in the Singaporean Malay Eye Study [34]. For visual impairment cutoff of 20/40, the occurrence of undercorrected refractive error was much higher, and almost two third of them (65.06%, 633/973) could experience a visual improvement of at least 2 lines with proper correction of their refractive errors. More than one forth of them (26.62%, 259/973) even gain four or more lines of visual acuity. In Tehran and Shihpai Eye studies [8,10], these two indicators were 76.1%, 62.0% and 88.2%, 48.0%, respectively, which was much higher than the results obtained in our study. In one study on Australian adults, undercorrected refractive errors were accountable for 56% of cases with visual impairment [35]. Correction of refractive errors also plays a role in the improvement of visual acuity of the uncorrectable visual impairment. In our study, 53.74% (201/374) of the subjects could experience a visual improvement in uncorrectable visual impairment, 12.57% (47/374) even gain four or more lines of improvement. All these results indicated that a significant proportion of visual loss was due to inadequately corrected refractive error and it is important and necessary to identify undercorrected refractive errors.

The definition of spectacle coverage refers to those who need visual correction and have proper spectacles. In order to represent the visual needs in modern life, e.g., driving, the cutoff of 20/40 was chosen. In spite of the increase in the number of spectacle users, there was still a large unmet need for spectacle correction as shown in this study and previous. The spectacle coverage varies in different regions, different ages and different ethnic groups, e.g., from 3.49% (Nigerian, ≥40 years) [36] and 25.2% (Bangladesh, ≥65 years) [37] to 71.2% (Taiwan, ≥65 years) [8] and 66.0% (Tehran, ≥5 years) [10]. The spectacle coverage in this study was 44.12%, which was consistent with those previous 4 studies. It was also found that spectacle coverage decreased as age increased. For example, the coverage was decreased from 56.99% in the subjects aged 60–69 years to 15.69% in the subjects aged≥80 years. Males in our study have a higher spectacle coverage than females (54.51% versus 36.47%), which was also consistent with previous reports [37].

Visual impairment is a global public health problem. Our study showed that visual impairment was significantly correlated with age, gender, occupation and the educational background. The occurrence of visual impairment in the subjects aged ≥80 years was 4-fold higher than that in the subjects aged 60–69 years. This age-related trend was consistent with previous population-based studies on the groups of subjects with different backgrounds [10,38] and educational status [10,38]. The observations that female had a significantly higher prevalence of visual impairment were also discovered in other regions of China [10]. This may be explained by Chinese traditional culture. In China, especially in rural areas, males gain more attentions than females in the family. Therefore, males have more opportunities of learning and employment, leading to a higher awareness of health care and higher quality of life. This culture can also explain why spectacles were more frequently worn by males than females.

In our study, 13.18% of participants had correctable visual impairment, 8.23% had uncorrectable visual impairment, and 77.73% had no impairment. These percentages were 7.5%, 3.6% and 88.9%, respectively, in the Blue Mountains Eye Study (age, ≥49 years) and were 5.6%, 2.7%, and 91.7%, respectively, in their 5-year follow-up studies [33]. The SEE Project (age≥65 years) reported that 5.6% of whites and 10.4% of blacks had correctable visual impairment [39]. In Shihpai Project (age≥65 years), the occurrence of correctable impairment was 9.6% [8]. Although our results were slightly different from other studies, which maybe due to the region and age of subjects, similar risk factors were identified: older age and female gender. Protective factors were higher level of education.

Visual impairment caused by refractive errors could be fully corrected using spectacle lens and the cost of spectacles is relatively low compared to eye surgery. Although sociodemographic factors, e.g., age, gender and education are important, it is still not sufficient to explain why the occurrence of correctable visual impairment is high and the spectacle coverage is low. The following reasons may explain the high occurrence of visual impairment and low spectacle coverage. Firstly, people are lacking the knowledge and awareness of refractive error. In a previous study of the SiMES population (n =503) on the knowledge of refractive errors, 26.6% (n =387) of subjects interviewed do not know that they have refractive errors [40]. Many senior people, particularly those with less educations, have a very common misunderstanding that loss of vision is naturally accompanied with increasing age, and nothing can be done to improve the situation [41,42]. In addition, peoplep with visual impairment refused to wear glasses because they were worried about the deterioration of the remaining vision. They thought that wearing glasses would increase the burden on the eyes, leading to the loss of their remaining eyesight. In such circumstance, health education is very necessary. It might also be the main reason why only a minority of our participants visited ophthalmic services, although the services are relatively easy to access in our city. Secondly, there is lack of regular visit to the eye care professionals because there are not enough ophthalmologist and refractionist compared with the increasing populations and the distribution of refraction services in China are not balanced between rural and urban areas [7,9,37,43]. Thirdly, some factors identified in the past, e.g., physically disabled, old age and frail, living alone, dependence on others for activities of daily life and travel inconvenience [32], may result in the consequence that these individuals are not exposed to the ophthalmic service. Fourthly, there are different requirement of life quality and cultures. Chinese people have the traditional virtues of patience. Meanwhile, some people have a relatively low demand of life quality and lower need of spectacles. This is an important reason that visual acuity in these adults remains uncorrected. Fifthly, the poor spectacle quality and the high cost of spectacles may also result in the visual impairment and low coverage of spectacles. One report indicated that approximately 50% of children in rural areas of China wear inappropriate glasses [44]. As the cost of refractive corrections, e.g., an appropriatespectacle, is still high compared with the personal and family resources in many regions in china and health insurance is not available in these regions, many people purchase their spectacles in some irregular glasses store.Optometric service must be accessible and affordable for people to reduce the growing occurrence of visual impairment.

Our results demonstrated that moderate/severe myopia had a higher spectacle coverage than mild myopia. The possible explanations for these findings are that near vision is maintained in mild myopic persons, although their far vision is seriously lost. Because the subjects in our study already retired, the lower rate of wearing glasses among mild myopia is due to less distant visual need. A lower rate of spectacle coverage was observed among hyperopia in our study, especially in mild hyperopia, which is different from the Bangladesh study showing that hyperopia were more likely to wear eyeglasses [37]. In this study, mild hyperopia showed significant higher spectacle coverage than moderate hyperopia. Mild hyperopia had once possessed relatively perfect vision in a long period of time. With growth of age and the decreasing accommodative capacity, both far and near vision are blurred. As their visual acuity experienced a process from clear to blur, these people tend to be more sensitive to changes in visual acuity and will seek medical help to achieve their need of visual acuity for daily activities. Because moderate hyperopia cannot get a clear far and near vision even at young age, these people normally give up treatment or thought that those refractive situations cannot be changed, very few people seek refractive corrections. With regard to the spectacle coverage of myopia and hyperopia, our results were consistent with the study in Tehran [10], showing that spectacle coverage was significantly higher in the myopia (72.6%) than that in the hyperopia (52.6%). Although the spectacle coverage in Tehran [10] was much high than our results, such differences may be explained by the differences in the age, because different age groups may have different need of visual acuity and the demand for life qualities. Different from the previous results, our studies showed that the spectacle coverage of people with astigmatism was the lowest. This is primarily due to the different definition of astigmatism used in our study. Astigmatism is defined as simple astigmatism without other refractive errors in the present study. Secondly, simple astigmatism has less severe vision loss. In addition, patients with astigmatism can reduce the visual interference and slightly improve eyesight by narrowing the palpebral fissure, frowning and head tilting. In summary, effect of refractive error on spectacle coverage need to be further investigated. Peasants have lower educational status and less regular ocular examinations. However, it is unexpected that the spectacle coverage in peasants is much higher than that in non- peasants. This might be associated with the unique historical background in this area. During the process of urbanization, the peasants in this area obtained generous government subsidies by land transfer. Therefore, the peasants in this area were wealthier than those in other areas and had the financial capacity to buy glasses. Peasants were also observed as protective factors for visual impairment in the present survey. We believe that our observation is a specific characteristic during the urbanization process in China. Further studies are needed to determine the underlying reasons.

## Conclusions

Our results demonstrated that a large number of visual impairment was caused by refractive error, but the spectacle coverage was relatively low. Attentions should be paid to the growing problems of visual impairment caused by refractive error. Public education, long-term community eye screening programs, primary refractive services, personnel training in refraction and other barriers that block the access to refractive correction should be identified in order to reduce the burden of refractive error in China.

## Abbreviations

BCVA: Best corrected visual acuity; SVI: Severe visual impairment; Mod VI: Moderate visual impairment; Mild VI: Mild visual impairment.

## Competing interests

The authors declare that they have no competing interests.

## Authors’ contributions

Mengjun Zhu participated in the design of this epidemiologic study, conducted and analyzed data in this study, contributed to the interpretation of the results and discussion, wrote and edited the manuscript. Xiaowei Tong ,Rong Zhao contributed to conception and design of this epidemiologic study, collected research data. Xiangui He, Huijuan Zhao,Meiling Liu participated in the research data collection and data analysis. Jianfeng Zhu participated in the design of this epidemiologic study, collected research data,contributed to the interpretation of the results and discussion and given final approval of the version to be published. All authors have read and approved the final manuscript.

## Pre-publication history

The pre-publication history for this paper can be accessed here:

http://www.biomedcentral.com/1471-2458/13/311/prepub
